# Oligosaccharides Modulate Rotavirus-Associated Dysbiosis and TLR Gene Expression in Neonatal Rats

**DOI:** 10.3390/cells8080876

**Published:** 2019-08-11

**Authors:** Ignasi Azagra-Boronat, Malén Massot-Cladera, Karen Knipping, Belinda van‘t Land, Sebastian Tims, Bernd Stahl, Jan Knol, Johan Garssen, Àngels Franch, Margarida Castell, Francisco J. Pérez-Cano, Maria J. Rodríguez-Lagunas

**Affiliations:** 1 Physiology Section, Department of Biochemistry and Physiology, Faculty of Pharmacy and Food Science, University of Barcelona (UB), 08028 Barcelona, Spain; 2 Nutrition and Food Safety Research Institute (INSA-UB), 08921 Santa Coloma de Gramenet, Spain; 3 Danone Nutricia Research, 3584 CT Utrecht, The Netherlands; 4 Division of Pharmacology, Utrecht Institute for Pharmaceutical Sciences, Faculty of Science, Utrecht University, 3584 CA Utrecht, The Netherlands; 5 University Medical Centre Utrecht/Wilhelmina Children’s Hospital, Department of Pediatric Immunology, 3584 EA Utrecht, The Netherlands

**Keywords:** rotavirus, microbiota, TLR, HMOs, scGOS/lcFOS

## Abstract

Colonization of the gut in early life can be altered through multiple environmental factors. The present study aimed to investigate the effects of 2’-fucosyllactose (2’-FL), a mixture of short-chain galactooligosaccharides/long-chain fructooligosaccharides (scGOS/lcFOS) 9:1 and their combination (scGOS/lcFOS/2’-FL) on dysbiosis induced during rotavirus (RV) diarrhea in neonatal rats, elucidating crosstalk between bacteria and the immune system. The dietary interventions were administered daily by oral gavage at days 2–8 of life in neonatal Lewis rats. On day 5, RV SA11 was intragastrically delivered to induce infection and diarrhea assessment, microbiota composition, and gene expression of Toll-like receptors (TLRs) in the small intestine were studied. All dietary interventions showed reduction in clinical variables of RV-induced diarrhea. RV infection increased TLR2 expression, whereas 2’-FL boosted TLR5 and TLR7 expressions and scGOS/lcFOS increased that of TLR9. RV-infected rats displayed an intestinal dysbiosis that was effectively prevented by the dietary interventions, and consequently, their microbiota was more similar to microbiota of the noninfected groups. The preventive effect of 2’-FL, scGOS/lcFOS, and their combination on dysbiosis associated to RV diarrhea in rats could be due to changes in the crosstalk between gut microbiota and the innate immune system.

## 1. Introduction

Microbial colonization is crucial for early life development of the gut by inducing maturation of the intestinal epithelium and gut-associated immune system [1]. The composition of commensal microorganisms, known as microbiota, is subject to dietary and environmental changes, driving the microbial ecosystem to adapt both qualitatively and quantitatively [2]. In healthy individuals, there is a balance in the gut microbiota, creating a homeostatic state inside the gastrointestinal tract which allows for a correctly functioning gut and limits overgrowth of potentially pathogenic microorganisms. Conversely, the term “dysbiosis” is used to define a state of altered microbiota composition which comprises structural imbalance as well as changes in the microbial functionality or metabolic activities [1]. Intestinal dysbiosis may include overgrowth of potentially harmful commensal members, reduction or complete loss of normally residing members, and loss of diversity [2,3].

The crosstalk between the immune system and the microbiota has a key role in shaping a healthy gut environment. The microbial sensing through pattern recognition receptors (PRRs), such as Toll-like receptors (TLRs) or Nod-like receptors (NLRs) is suggested to influence microbial colonization and correct immune development [2,4]. Moreover, PRRs play an essential role in the innate immune system by recognizing microbe-specific and damage-associated molecules, by triggering host defensive responses, and by providing protection from invading intestinal pathogens. The ligands that activate PRR signaling pathways are well-known, for example, lipopolysaccharide activates TLR4 and flagellin activates TLR5 [5]. When this preventive mechanism fails to contain or prevent the infection, pathogens can trigger excessive inflammation, thereby altering the composition of the microbiota (dysbiosis) and the barrier function, as seen in animal models [6].

Group A rotaviruses (RVs) are the main cause of dehydrating diarrhea in children under 5 years of age worldwide, contributing up to 130,000 deaths annually [7]; indeed, almost every child becomes infected at least once in their first 5 years of life [8]. There is few evidence supporting microbiota alterations due to RV infection; to date, this link has been suggested at the preclinical level in piglets [9,10] and in humans [11,12]. Furthermore, the microbiota composition can modulate RV infection and have an impact in the susceptibility of the disease [13] or in response to RV vaccination [14,15,16,17]. Changes in the microbiota, for example, by the use of antibiotics, can also modulate RV infection or immunization [18,19].

There is increasing interest in the potential of oligosaccharides with prebiotic activity for both the prevention and treatment of infectious diseases. On the one hand, oligosaccharides exert their anti-infective activity by stimulating the composition and/or activity of certain commensal bacteria found in the gut. These changes can induce a broad array of effects, such as an increase of the production of antimicrobial peptides, the competition for an available ecological niche, or an increase in short-chain fatty acid (SCFA) production, leading to lower luminal pH and higher mucin production, thereby contributing to exclude pathogen invasion [20,21,22]. On the other hand, some oligosaccharides are considered to have other anti-infective activities independent from those exerted on the commensal bacteria, such as direct interactions with the immune system, with the intestinal epithelium, or with the pathogens themselves [23]. Within the complex composition of human milk oligosaccharides (HMOs), the globally most abundant HMO, 2’-fucosyllactose (2’-FL), has promising effects in vitro and in vivo by reducing the infectivity or by preventing diarrhea induced by some pathogens, including RV [10,24,25,26,27] as well as improving vaccination responses in vivo [28]. In addition, a mixture of short-chain galactooligosaccharides (scGOS) and long-chain fructooligosaccharides (lcFOS) at a proportion of 9:1 has also shown benefits on RV-induced diarrhea in early life [10,27,29,30]. There are few published studies in regard to the effects of 2’-FL and/or scGOS/lcFOS on human microbiota composition in healthy conditions [31,32,33] and even less in the context of RV infection; only preclinical data is available in a piglet model [10]. On the other hand, it has been recently described the importance of the interaction between HMOs and microbiome of milk, as well as neonatal microbiome on rotavirus infection [34].

Therefore, we aimed to investigate the effects of 2’-FL, scGOS/lcFOS, and their combination on the dysbiosis caused by RV diarrhea in neonatal rats. Moreover, changes in the bacteria–host crosstalk that could be linked to microbiota composition and oligosaccharides anti-infective effects were studied.

## 2. Materials and Methods

### 2.1. Animals

Fifteen G15 pregnant Lewis rats (LEW/OrlRj) were provided by Janvier Labs (Le Genest Saint Isle, France), individually housed in cages (2184L Eurostandard Type II L, Tecniplast, West Chester, PA, USA) with large fibrous particles bedding (Souralit 1035, Bobadeb S.L., Santo Domingo de la Calzada, Spain) and tissue papers (Gomà-Camps S.A.U., La Riba, Spain), and monitored daily. After natural delivery, litters were randomly assigned to the experimental groups and culled to 8 pups per lactating dam, with a similar number of females and males in each litter. Dams received a commercial diet (Teklad Global Diet 2014, Envigo, Indianapolis, IN, USA) and water ad libitum. Animal handling was performed during the first hours of the light phase on a scheduled basis to limit the disturbance and influence of biological rhythms. After separating all the mothers and keeping the pups in the home-cage, handling and oral administration was performed. Afterwards, the dam was reunited with the whole litter. They were housed under controlled conditions of light (12 h light–12 h dark cycle), temperature, and humidity in a biosecurity level 2 isolated room in the Faculty of Pharmacy and Food Science animal facility (University of Barcelona, Spain). All experimental procedures were approved by the Ethical Committee for Animal Experimentation of the University of Barcelona and the Catalonia Government (CEEA-UB Ref.74/05, DAAM: 3046).

In regard to sample size estimation, three litters were required for each group as previous studies have demonstrated a remarkable variability among litters [30]. The Appraising Project Office’s program from the Universidad Miguel Hernández de Elche (Alicante) was used to calculate the minimal sample size to provide statistically significant differences among groups. The final number of animals was not affected by the dropouts or outliers, which did not occur in the present study.

### 2.2. Experimental Design

The present study is part of a larger project (“Preventive antidiarrhoeic effect of scGOS/lcFOS and 2-FL on a rat neonatal RV infection model”) in which neonatal rats were given the same oligosaccharides from the second to the sixteenth day of life, aiming to evaluate global clinical outcomes and modulation of the immune response throughout and at the end of the diarrheic period [27]. Herein, the study focuses on the effect of the oligosaccharides on the fecal microbiota composition at the peak of the diarrhea induced by RV, corresponding to day 8 of life.

scGOS/lcFOS and 2-FL were selected for the study because they are oligosaccharides already added to infant formula with anti-infective and immunomodulatory effects both at the preclinical and human levels [24,25,26,27,28,29,30].

Upon natural delivery, newborn rats were randomly distributed into five groups depending on the supplementations given (provided by Danone Nutricia Research, Utrecht, The Netherlands)—the reference (REF), rotavirus-infected (RV), and rotavirus-infected—and that received: a) a mixture of scGOS and lcFOS in a 9:1 ratio (RV+scGOS/lcFOS); b) 2’-FL (RV+2’-FL); and c) both scGOS/lcFOS and 2’-FL (RV+scGOS/lcFOS/2’-FL). The analyses of the oligosaccharides were performed by either high performance anion exchange chromatography (HPAEC) or liquid chromatography-mass spectrometry (LC-MS) as previously described [35,36].

Neonatal rats were orally administered from the second to the eighth day of life, when the diarrhea reaches its maximum score, as previously described [29]. The RV+scGOS/lcFOS group was given 0.8 g of scGOS/lcFOS per 100 g of body weight (4.5 µL/g/day), the RV+2’-FL group was given 0.2 g of 2’-FL/100 g of body weight (4.5 µL/g/day), and the RV+scGOS/lcFOS/2’-FL group received both products at the same doses as when administered separately (4.5 µL/g/day). A matched volume of water was administered to the animals from the REF and RV groups. 2’-FL was produced by microbial fermentation, with >90% purity. The selected dose of GOS/FOS was based on previous studies in similar approaches [29] and that of 2-FL was based on the daily consumption of an infant of this particular HMO per body weight [37,38] and on previous studies [27,39]. In addition, it has been demonstrated to be a safe dose in rats [40].

The RV (simian SA-11) was obtained as previously described [41] and inoculated at day 5 of life (4 × 10^8^ Tissue Culture Infectious Dose 50 [TCID50]/rat) in all the experimental groups with the exception of the REF group, which received the same volume of PBS under the same conditions. At day 8 of life, 3 days after RV inoculation, half of each litter (4 random pups, 3 litters/group, n = 12) were euthanized to obtain tissue samples for the current study.

### 2.3. Clinical Evaluation and Sample Collection

Fecal sampling was performed once daily throughout the study (from day 4 to day 8 of life) by gently pressing and massaging the abdomen. Severity of diarrhea was expressed by fecal weight and by scoring fecal samples from 1 to 4 (diarrhea index, DI) based on color, texture, and amount as follows: normal feces (1); soft yellow-green feces (2); totally loose yellow-green feces (3); and high amount of watery feces (4). Sample scoring was performed by two senior investigators in a blinded manner. Scores ≥ 2 indicate diarrheic feces, whereas scores < 2 indicate absence of diarrhea [41]. Fecal samples were weighed and stored **at** −80 °C until the analysis of the microbiota composition.

Incidence of diarrhea was expressed by the percentage of diarrheic animals (%DA), which is based on the percentage of animals displaying scores of DI ≥ 2 in each group. The DI and %DA were normalized in the groups receiving scGOS/lcFOS and scGOS/lcFOS/2’-FL because of intrinsic effects on the fecal consistency of each supplementation. For the normalization, the mean DI was calculated for the timepoints when there was no active diarrhea in the RV group (before the infection on days 4–5 and after the diarrhea was resolved on days 12–16). The difference between this mean and the baseline score of DI = 1 was subtracted to all values of DI for RV+GOS/FOS and RV+GOS/FOS+2’-FL groups.

At day 8 of life, neonatal rats were intramuscularly anesthetized with ketamine (90 mg/kg) (Merial Laboratories S.A., Barcelona, Spain) and xylazine (10 mg/kg) (Bayer A.G., Leverkusen, Germany) and exsanguinated. A section of 1 cm of the central part of the small intestine was collected in RNA later for the gene expression analysis.

### 2.4. Quantification of Gene Expression by Real-Time PCR

As previously described [42], the small intestine, including duodenum, jejunum, and ileum, was obtained and 1 cm portion of the central section of the whole intestine was sectioned. It was homogenized during 30 s in lysing matrix tubes (MP Biomedicals, Illkirch, France) using a FastPrep-24 instrument (MP Biomedicals). RNA was isolated with the RNeasy® Mini Kit (Qiagen, Madrid, Spain) following the manufacturer’s instructions. RNA purity and concentration were determined with a NanoPhotometer (BioNova Scientific S.L., Fremont, CA, USA). Later, cDNA was obtained in a thermal cycler PTC-100 Programmable Thermal Controller using TaqMan® Reverse Transcription Reagents (Applied Biosystems, AB, Weiterstadt, Germany).

The specific PCR TaqMan® primers (AB) used to assess gene expression with real-time PCR (ABI Prism 7900 HT, AB) were *Tlr2* (Rn02133647_s1, inventoried, I), *Tlr3* (Rn01488472_g1, I), *Tlr4* (Rn00569848_m1, I), *Tlr5* (Rn04219239_s1, I), *Tlr7* (Rn01771083_s1, I), and *Tlr9* (Rn01640054_m1, I). The relative gene expression was normalized with the housekeeping gene *Gusb* (Rn00566655_m1, I) using the 2-ΔΔCt method [43]. The results are expressed as the percentage of expression in each experimental group normalized to the mean value obtained for the REF group, which was set at 100%.

### 2.5. Analysis of Fecal Microbiota Composition by 16S rRNA Sequencing

A representative animal from each litter was included in the microbiota composition analysis (3 litters/group, n = 3). For that, the selected animal in each litter met the following criteria: being an animal with a fecal sample collected on day 8 and not being one of the animals with the highest or the lowest DI scores. Genomic DNA was extracted from the fecal samples ranging 5–20 mg collected on day 8, using the Qiamp DNA Stool Mini kit (Qiagen) with some previous modification, enzymatic lysis, and mechanic disruption [44]. Extra purification and concentration were performed following the cleaning protocol from the Qiamp Micro kit (Qiagen). DNA concentrations obtained were 6.6 ng/μL (from 1.3 to 22.3 ng/μL) with purities of 1.89 for A_260/280_ and 0.36 for A_260/230_. In all cases, enough DNA was obtained and 50 ng of DNA were amplified following the 16S Metagenomic Sequencing Library Illumina 15044223 B protocol (Illumina Inc, San Diego, CA, USA) in collaboration with Lifesequencing S.L (Valencia, Spain). In brief, in the first amplification step, primers were designed containing a universal linker sequence allowing amplicons for incorporation indexes, sequencing primers by Nextera XT Index kit (Illumina Inc.), and 16S rRNA gene universal primers for the V3–V4 regions [45]. In the second and last amplifications, indexes were included. Libraries were quantified by fluorimetry using the Quant-iT™ PicoGreen™ dsDNA Assay Kit (Thermo Fisher Scientific) and pooled prior to sequencing on the MiSeq platform (Illumina Inc.), with a configuration of 300 cycle paired reads. The size and quantity of the pool were assessed in the Bioanalyzer 2100 (Agilent) and with the Library Quantification Kit for Illumina (Kapa Biosystems Inc., Wilmington, MA, USA), respectively. PhiX Control library v3 (Illumina Inc.) was combined with the amplicon library (expected at 20%). Sequenced data were available within approximately 56 h. Image analysis, base calling, and data quality assessment were performed in the MiSeq instrument.

The software Paired-End read merger (PEAR v 0.9.6, Exelixis Lab, Heidelberg, Germany) was used to merge raw sequences forward and reverse in order to obtain the complete sequence. Using this approach, the ends of the obtained sequences were overlapped in order to get complete sequences. The amplification primers from the sequences obtained in the sequencing step were trimmed with Cutadapt v1.8.1 [46], using parameters by default, in order to reduce the bias in the annotation step. Once the primers had been removed, sequences lower than 200 nucleotides were excluded from the analysis because short sequences have a higher chance to generate wrong taxonomical group associations.

After obtaining the clean complete sequences, a quality filter was applied in order to delete sequences with poor quality. Those bases in extreme positions that did not have Q20 (99%) of well-incorporated bases in the sequencing step or more Phred quality score were removed, and later, sequences of which quality means did not surpass the Q20 threshold, as a mean quality of the whole sequence, were also deleted. The resulting sequences were inspected for PCR chimera constructs that may occur during the different experimental process, which were removed from further analysis. Each group of sequences was compared to a database of rRNA using an alignment BLAST strategy to associate taxonomic groups. The relative proportions of phyla, families, genus, and species were calculated. Moreover, to estimate the specific biodiversity, the Shannon Index and CHAO1 indexes were calculated.

To visualize the presence or absence of certain bacterial genera and species in the experimental groups, the criteria used were all bacterial groups detected in 2 or 3 animals with proportions higher than 0.001% were regarded as present, while the bacterial groups detected in just 1 animal or none were regarded as absent. On that basis, the Venn diagrams were constructed to visualize numerically how the genera and species were distributed among the groups in order to compare their coincidences and differences. Furthermore, the log2 fold change of all genera and species was calculated with respect to the RV group and were represented in heat maps. Finally, sequences that closely resemble bacterial strains with accepted probiotic activity [47] were selected and represented in a heat map by calculating their relative percentage in each group, where the group with the highest bacterial count was set at 100% and the group with the lowest bacterial count at 0%.

### 2.6. Principal Components Analysis

Principal Components Analysis (PCA) was conducted to evaluate the dimensionality of microbiota profile with regard to the infection and oligosaccharide supplementations. To develop the model, Simca v14.1 was used (Umetrics, Umeå, Sweden). Two data matrices were constructed consisting of 15 rows (15 samples/group) and 100 variables corresponding to the taxonomic analysis of genera or 189 variables corresponding to the taxonomic analysis of species. PCA was conducted on both data matrices in order to explore the presence of any natural clustering in the data. In the preprocessing of the PCAs, the mean-centering and unit variance scaling were applied.

### 2.7. Statistical Analysis

The Statistical Package for the Social Sciences (SPSS v22.0) (IBM, Chicago, IL, USA) was used for statistical analysis. Data was tested for homogeneity of variance and normality distribution by the Levene’s and Shapiro–Wilk tests, respectively. When data was homogeneous and had a normal distribution, conventional one-way ANOVA test followed by the post-hoc Bonferroni was performed. Otherwise, the nonparametric Kruskal–Wallis test followed by the post-hoc Mann–Whitney U test were performed. Due to the limited sample size, the distribution of the resulting *p*-values was such that it did not allow proper multiple testing correction. The Chi-square test was used to compare frequencies of diarrhea incidence (%DA). Finally, correlations of clinical data with TLRs gene expression and microbiota levels were performed by calculation of the Pearson’s correlation coefficient. Differences were considered significant when *p* < 0.05.

## 3. Results

### 3.1. Clinical Assessment

**Figure 1:**
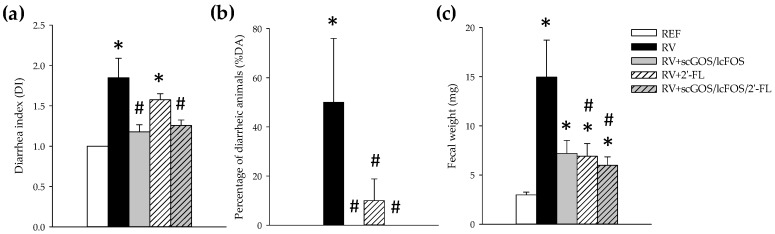
Clinical variables of severity and incidence of diarrhea on day 8: (**a**) The Diarrhea Index (DI) is based on scoring fecal samples from 1 to 4 depending on the color, texture, and abundance. Scores of DI ≥ 2 indicate the presence of diarrhea, whereas scores < 2 indicate the absence of diarrhea. (**b**) The percentage of diarrheic animals (%DA) is based on the percentage of animals displaying DI scores ≥ 2 in each group. (**c**) The mean fecal weight was calculated in each group as an objective indicator of diarrhea severity. The results are expressed as mean ± S.E.M. (n = 8–12/group, depending on the number of fecal samples obtained) * *p* < 0.05 compared to the reference (REF) group; # *p* < 0.05 compared to the RV group ((a) by ANOVA; (b) by the Chi Squared test; and (c) by the Mann–Whitney U test).

RV inoculation in rats induced a moderate acute diarrhea, displaying the highest affectation at day 8 of life (3 days after RV inoculation). The severity of diarrhea, calculated as the DI (Figure 1a), was significantly reduced for both RV+scGOS/lcFOS and RV+scGOS/lcFOS/2’-FL (*p* < 0.05 vs. RV), whereas although the 2’-FL group displayed a lower severity, no statistical differences were seen as compared to the control RV infection. Moreover, when calculating the percentage of diarrheic animals (%DA) as an indicator of incidence (Figure 1b), all three supplementations were able to profoundly reduce the proportion of animals displaying diarrhea (DI ≥ 2); indeed, none of the animals in the RV+scGOS/lcFOS and RV+scGOS/lcFOS/2’-FL groups displayed diarrhea and only 10% in RV+2’-FL group did. Although on day 8 only 50 % of the animals displayed diarrhea in the RV group, almost all animals (21/24, 87.5%) displayed DI ≥ 2 at some timepoint of the diarrhea process because some of them reached the peak of the disease on days 6 or 7. In fact, the day in which the maximum score was obtained was 7.54 ± 0.20 for the RV group, 7.40 ± 0.87 for the GOS/FOS group, 6.83 ± 0.32 for the 2-FL group, and 6.25 ± 0.25 for the group receiving the combination of both products. The fecal weight, as an objective indicator of the disease, increased due to RV diarrhea. However, such increase was prevented by supplementation with all the oligosaccharides (Figure 1c), being statistically different for the RV+2’-FL and RV+scGOS/lcFOS/2’-FL groups (*p* < 0.05 vs. RV group).

### 3.2. Intestinal Toll-Like Receptors Expression

**Figure 2:**
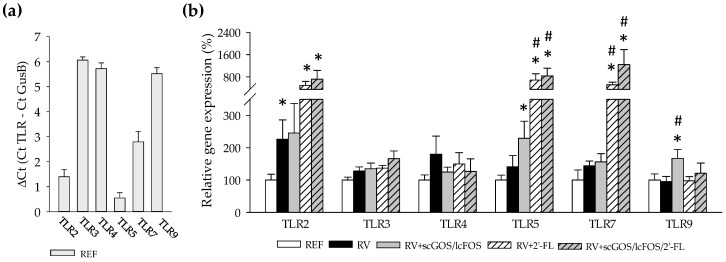
Small intestine gene expression of Toll-like receptors (TLRs) at the peak of diarrhea (day 8): The transcription of TLR2, 4, 5, 7, and 9 were quantified by real-time PCR. (**a**) The ∆Ct of TLR expression compared to *Gusb* was calculated in REF animals. (**b**) The relative gene expression in the experimental groups was calculated with respect to REF, which corresponded to 100% of transcription. Results are expressed as mean ± S.E.M. (n = 8/group). * *p* < 0.05 compared to the REF group; # *p* < 0.05 compared to RV group (by the Mann–Whitney U test).

In order to better understand the role of oligosaccharide supplementation in the immune response and its crosstalk with the microbiota in the context of RV infection, the gene expression of Toll-like receptors (TLRs) was assessed by real-time PCR (Figure 2). The highest expression in REF rats was found in TLR3, TLR4, and TLR9 (Figure 2a). RV infection did not alter considerably the gene expression of the TLRs studied, with exception of TLR2, which was increased in the RV group (*p* < 0.05, Figure 2b). Similarly, the dietary supplementations with 2’-FL and its combination with scGOS/lcFOS increased other TLRs gene expression compared to the REF and RV groups, such as TLR5 and TLR7 (*p* < 0.05). In addition, rats receiving scGOS/lcFOS also increased significantly the expression of TLR9 (*p* < 0.05 vs. the RV and REF groups). The expressions of TLR3 and TLR4 were not modified either by the infection or the supplementations. Despite that, TLR4 gene expression positively correlated with the DI when clustering all RV-infected animals together (r = 0.422, *p* < 0.05).

### 3.3. Fecal Microbiota Composition

#### 3.3.1. Overall Diversity

The fecal microbiota composition was assessed during the peak of diarrhea in order to ascertain whether the RV infection or the supplementation with oligosaccharides in the presence of RV were able to alter the microbial populations. No differences were observed in the number of sequences and Operational Taxonomic Units (OTUs, Table 1). Moreover, the Shannon Index and the CHAO1, indicating diversity and richness of the microbial community, respectively, were similar among groups (Table 1).

#### 3.3.2. Taxonomic Analysis

**Figure 3:**
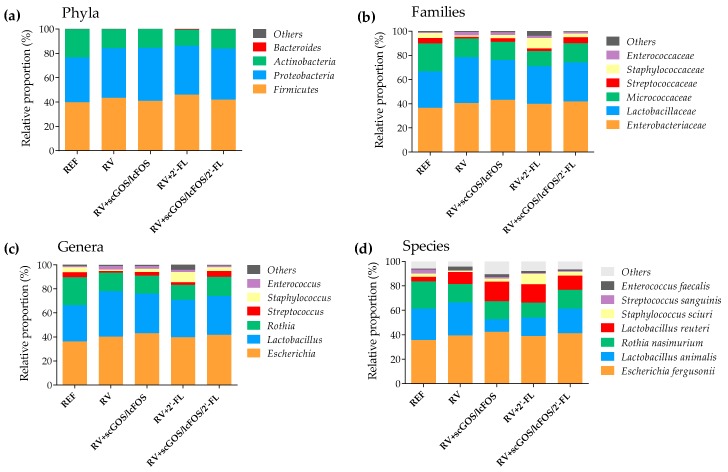
Main taxonomic ranks showing the proportion of bacterial populations in the fecal content at the peak of diarrhea (day 8): The sequencing of the amplicon targeting the V3–V4 region of the 16S rRNA was performed following the 16S Metagenomic Sequencing Library Illumina 15044223 B protocol. The relative proportion of the bacteria was calculated in each taxonomic rank: (**a**) phylum, (**b**) family, (**c**) genus, and (**d**) species. The results are expressed as mean (n = 3/group). Significant differences are shown only in the text (by the Mann–Whitney U test).

The taxonomic analysis of the microbial populations showed that *Firmicutes* and *Proteobacteria* were the most abundant phyla in the neonatal rat fecal microbiota (Figure 3a). RV infection did not change the proportion of the different phyla, although a tendency to reduce microorganisms from the *Actinobacteria* phylum was detected in all RV-infected groups compared to REF. The supplementations with oligosaccharides did not show relevant differences either, with the exception of increasing the *Proteobacteria* proportion in the scGOS/lcFOS group (*p* < 0.05 vs. REF).

In contrast, the family abundance of the neonatal rat fecal microbiota was notably changed by both the RV infection and the oligosaccharides supplementations (Figure 3b). The RV group decreased the proportion of *Streptococcaceae* with respect to REF (*p* < 0.05), which was prevented by the three supplementations. Indeed, the supplementation with scGOS/lcFOS and scGOS/lcFOS/2’-FL had a higher proportion of this family than the RV group (*p* < 0.05). On the other hand, the RV group showed a trend to reduce the members of the *Staphylococcaceae* family compared to the REF group, whereas the administration of scGOS/lcFOS and 2’-FL increased their proportion in comparison with the RV group (*p* < 0.05).

The analysis of microbiota at the genus level (Figure 3c) did not show notable changes in *Lactobacillus* or *Escherichia*, which totaled more than 60% of the neonatal fecal population. Nevertheless, the RV+2’-FL and RV+scGOS/lcFOS/2’-FL displayed a reduction in the proportion of the *Rothia* genus (the third most abundant) compared to the REF group (*p* < 0.05).

Interestingly, the lower proportion of *Streptococcaceae* by RV infection caused a decrease in *Streptococcus* which was prevented by scGOS/lcFOS and scGOS/lcFOS/2’-FL supplementations (*p* < 0.05). Similarly, the decrease in *Staphylococcaceae* was due to the reduction in the proportion of *Staphylococcus* in the RV group which was counteracted by the supplementation with scGOS/lcFOS and 2’-FL administration.

The supplementations also induced changes in minor populations compared to both the RV and REF groups (Figure 3c, included in the section termed “others”). All oligosaccharides showed an increase in genera belonging to the *Firmicutes* and *Actinobacteria* phyla. For example, scGOS/lcFOS and its combination with 2’-FL increased *Turicibacter* compared to the REF and RV groups (0.077 ± 0.032% and 0.039 ± 0.023% vs. 0.005 ± 0.001% and 0.002 ± 0.001%, respectively; *p* < 0.05). Moreover, scGOS/lcFOS had a tendency to increase *Bifidobacterium* compared to the REF and RV groups (0.194 ± 0.194% vs. 0.014 ± 0.011% and 0.006 ± 0.003%, respectively). This trend towards an increase in *Bifidobacterium* was even higher in the animals receiving 2’-FL (up to 0.555 ± 0.283%).

More than 90% of the microbiota composition comprised 7 species (Figure 3d). All RV-infected groups tended to increase the levels of *Enterococcus faecalis*, which only attained statistical significances in the RV+scGOS/lcFOS group (*p* < 0.05 vs. REF). Moreover, all RV-infected groups reduced the levels of *Streptococcus sanguinis* compared to the REF group (*p* < 0.05). The supplementations induced other specific changes, such as a decrease in the proportion of *Lactobacillus animalis* in the RV+scGOS/lcFOS and RV+scGOS/lcFOS/2’-FL groups compared to both the REF and RV groups (*p* < 0.05). Moreover, animals administered only with scGOS/lcFOS showed an increase in the proportion of *Lactobacillus reuteri* (*p* < 0.05 vs. REF).

**Figure 4:**
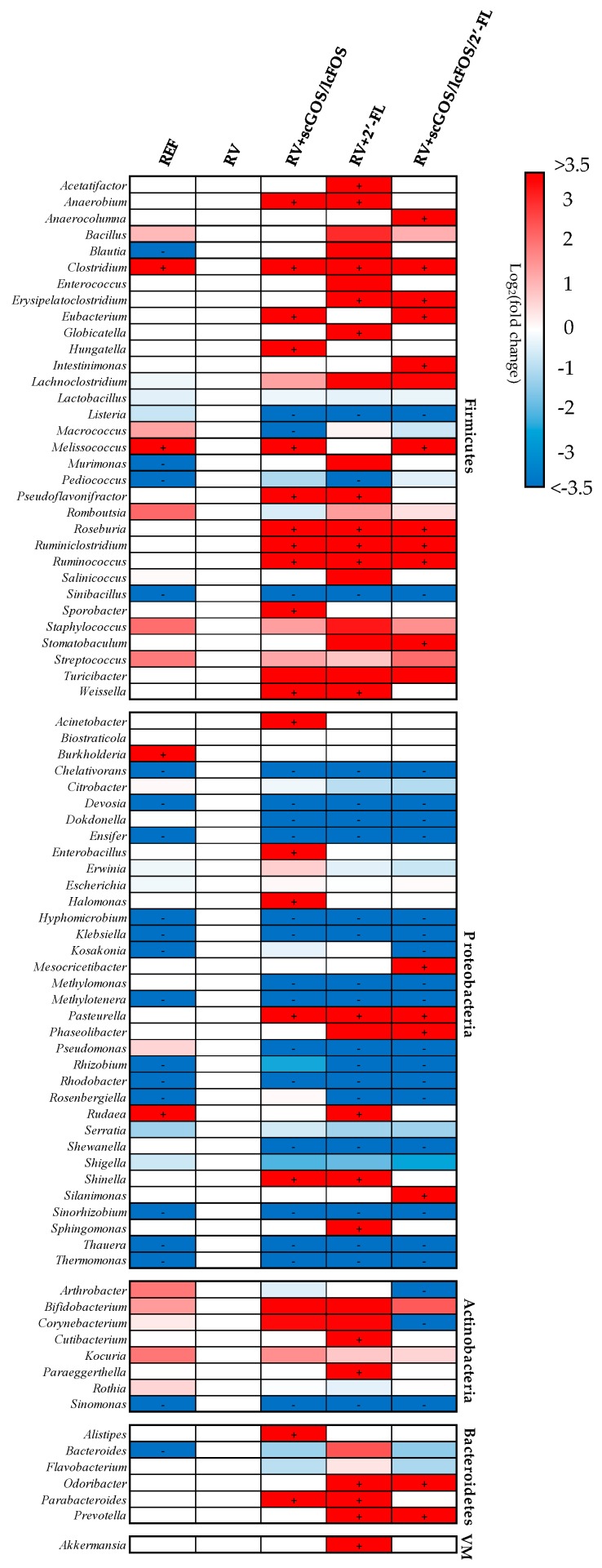
Summary of genera variation in fecal microbiota at the peak of diarrhea (day 8): A heat map of the mean relative abundances of the prominent OTUs assigned to the genus level is represented. The rows represent the genera and the columns the experimental groups. The log2 of the fold change with respect to the RV group was calculated and assigned a color following the legend. Genera which were present in a group but not in the RV group were assigned the maximum variation (>3.5, marked with a “+”), whereas the genera which were presented in the RV group and not in the other groups were assigned the minimum variation (<−3.5, marked with a “-“). The results are derived from n = 3/group. VM: *Verrucomicrobia*.

In order to better visualize and combine both the qualitative and quantitative proportions of the genera and species present in feces, two heat maps were created (Figure 4 and Figure S1). Hence, it was found that RV infection promoted the colonization of genera from the *Proteobacteria* phylum (e.g., *Hyphomicrobium*, *Klebsiella*, or *Rhodobacter*), which were not detected in the REF group. Moreover, most of these genera were not found either in the groups administered with scGOS/lcFOS, 2’-FL or the combination of both. In addition, concerning *Firmicutes* phylum, *Clostridium* was not detected in the RV group but it was detected in all the other groups.

Correlation analysis between microbiota, clinical outcomes, and TLRs was also performed. The DI positively correlated with 8 genera (*p* < 0.05, *Chelativorans* (r = 0.958), *Hyphomicrobium* (r = 0.903), *Klebsiella* (r = 0.958), *Methylotenera* (r = 0.968), *Pediococcus* (r = 0.813), *Rhodobacter* (r = 0.834), *Sinibacillus* (r = 0.958), and *Thauera* (r = 0.958)), which were exclusively found in the RV group with the exception of *Pediococcus*, which was also present in the groups administered with scGOS/lcFOS and scGOS/lcFOS/2’-FL. However, it should be taken into account that these genera were not abundant because their proportions in the samples were lower than 0.005%. Moreover, the correlations between microbiota and TLRs were also analyzed gathering all the infected groups. Although 30 genera correlated with the expression of some TLRs, 28 of them were with genera poorly represented in the samples (<0.01%). From the remaining genera, negative correlations between the proportion of *Lactobacillus* and TLR5 (r = −0.755, *p* < 0.05) and of *Rothia* and TLR9 (r = −0.921, *p* < 0.05) were found.

#### 3.3.3. Venn Diagrams and Principal Components Analysis: Genera and Species

**Figure 5:**
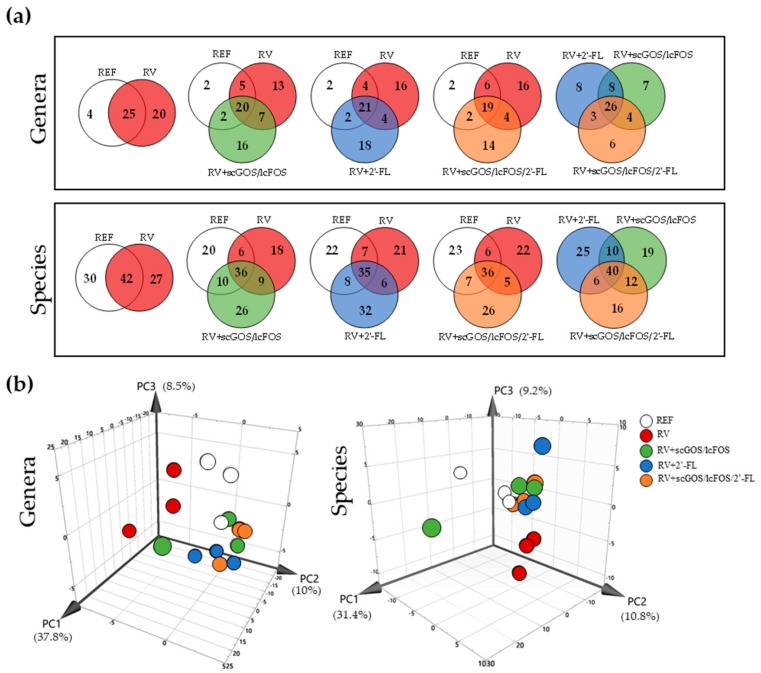
Analysis of the fecal microbiota diversity in genera and species at the peak of diarrhea (day 8): (**a**) Representation of Venn diagrams and (**b**) Principal Components Analysis (PCA) of all genera and species. To estimate the presence or the absence of these taxa in the experimental groups, it was agreed that all groups displaying 2 or 3 animals (out of the 3 analyzed in each group) with a bacterial proportion were computed as present, while the groups displaying 1 animal or none were computed as absent. The results are derived from n = 3/group.

The impact of RV infection and oligosaccharides supplementation on fecal microbiota genera and species was further assessed by an analysis of Venn diagrams and PCAs (Figure 5). Venn diagrams allowed the observation of these differences numerically (Figure 5a). This approach could possibly reduce the background variability represented by transient or subject-specific microbes, allowing the better characterization of those changes due to RV infection and oligosaccharides supplementation.

There was a core of 19–21 bacterial genera, which persisted in all three groups and accounted for more than 95% of the microbiota. Comparing the microbiota of the REF and RV groups at the genus level, a remarkable qualitative alteration of the fecal microbiota due to the infection can be observed. The RV caused a dysbiosis observed by the colonization of 20 new genera (e.g., *Klebsiella*, *Pediococcus*, and *Rhizobium*) while 4 of the genera present in the REF group were not detected (*Burkholderia*, *Clostridium*, *Melissococcus*, and *Rudaea*).

In regard to the supplementations, several of those genera that appeared due to the infection (13–16 out of the 20 in the RV group) were not detected after the oligosaccharide interventions. Instead, 14–18 new genera, which were absent in either the RV or the REF groups, colonized the intestine (e.g., *Pasteurella*, *Roseburia*, and *Ruminococcus*).

The analysis of species with the Venn diagram approach showed similar results, maintaining a core of 35–42 species in all groups as the main bacterial populations independently of the infection and supplementations. In addition, several RV- and supplementation-specific species that colonized the intestine were modified (Figure 5a).

PCAs were plotted (Figure 5b) in order to reduce the dimension of these taxonomic data while retaining the maximum variation (a cumulative 56.3% and 51.4% on PC1 until PC3 for genera and species, respectively). The microbiota of the RV group clearly clustered apart from those of the REF group and the oligosaccharide-receiving groups infected with RV in both the analysis of genera and species. In contrast, the REF animals and those receiving oligosaccharides clustered together in the case of species but not so clearly in the case of genera because the supplementations seemed to induce some changes that clustered them apart from 2 individuals of the REF group.

#### 3.3.4. Assessment of Prebiotic Potential of Oligosaccharides

**Figure 6:**
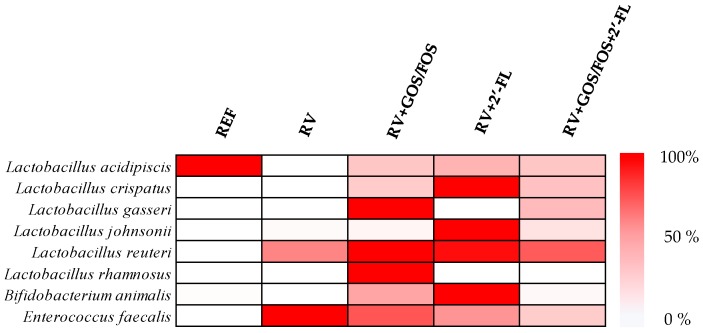
Analysis of the main bacterial species with probiotic activity found in the fecal microbiota at the peak of diarrhea (day 8): The heat map displays the relative abundance of bacteria with probiotic activity, where the group with the highest abundance was set at 100% and the group with the lowest abundance was set at 0%. The results were derived from n = 3/group.

The prebiotic activity of the oligosaccharides was also assessed by observing the relative abundance in selected bacterial species that have been attributed probiotic properties (Figure 6). All supplementations induced a clear prebiotic effect, as seen by higher relative counts of these beneficial microorganisms. In particular, scGOS/lcFOS supplementation induced the highest prebiotic effect promoting the growth of *Lactobacillus gasseri*, *Lactobacillus reuteri*, *Lactobacillus rhamnosus*, and *Bifidobacterium animalis*, whereas 2’-FL induced an increase in *Lactobacillus crispatus*, *Lactobacillus johnsonii*, *Lactobacillus reuteri*, and *Bifidobacterium animalis* populations. No correlations between the proportion of these species, and the DI were observed.

## 4. Discussion

**Figure 7:**
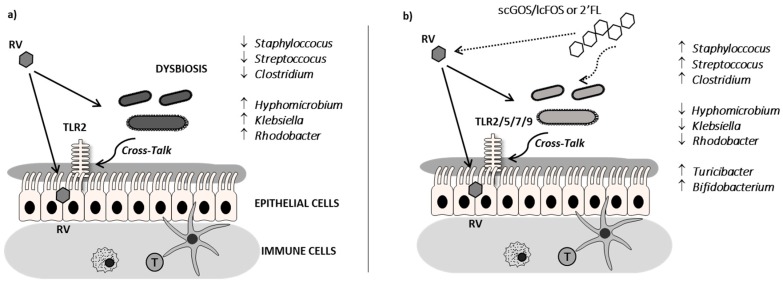
Summary of the effects of the RV and the scGOS/lcFOS and 2’-FL on microbiota composition and crosstalk: (**a**) Microbial imbalance produced by the RV infection in the neonatal rat model in terms of crosstalk alterations and microbiota composition with respect to a noninfected group (REF) and (**b**) Preventive effects of scGOS/lcFOS and 2’-FL on the dysbiosis caused by the RV.

Throughout early life, virtually all infants are exposed to RV infection, leading to vomiting, fever, and diarrhea. In this study, oligosaccharide supplementations have shown beneficial immunomodulatory effects, such as the increase in TLR expression, which we hypothesize could indicate a better sensing and clearance of the RV. In addition, we have shown for the first time that the challenge with RV SA11 in suckling rats induces intestinal dysbiosis and that this disturbed microbiota can be prevented by the administration of certain oligosaccharides (Figure 7).

The challenge with RV in 5-day suckling rats induced moderate diarrhea, which displayed the highest clinical symptoms at 3 days postinoculation (day 8), when the incidence was 50% in the RV group.

The present results demonstrate that both 2’-FL and GOS/FOS, as well as their combination, displayed amelioration in terms of the severity and incidence of RV infection. These results agree with previous studies in our group [27,29,30,48]. In regard to the mechanisms by which oligosaccharides can prevent infections, they might involve both microbiota-independent and microbiota-dependent actions [49]. Our previous studies were focused on microbiota-independent mechanisms, the direct effect on the virus, and the gut epithelial and immune cells, evidencing that the mechanisms involved were different between both oligosaccharides. 2’-FL displayed a higher direct ability to promote intestinal maturation and to enhance neonatal immune responses, whereas GOS/FOS induced an intestinal trophic effect and a higher RV-blocking action, and their combination showed additive effects in some variables [27]. These effects are in line with the prevention of pathogen binding and the modulation of intestinal epithelial and immune cell response effects ascribed to certain HMOs [37]. Besides this, the present study has focused on the microbiota-dependent mechanisms of these oligosaccharides by means of the study of the TLR gene expression as a first approach to evaluate bacteria-host interaction and the study of their ability to modulate gut microbiota composition. Some oligosaccharides have been attributed immunomodulatory activities regarding TLR functionality, as they may act directly as TLR ligands or indirectly, for example, by changing the microbiota and thus the immunoreactive profile [4,50,51,52,53]. The gene expression of several intestinal TLRs was boosted by the RV infection and the supplementations. On the one hand, the challenge with RV induced a higher expression of TLR2 in all groups (with exception of the RV+scGOS/lcFOS group, which only displayed a trend to increase). This fact could be explained by considering the ability of TLR2 to recognize RV Non-Structural Protein 4 (NSP4), which is a glycoprotein playing an essential role in the RV assembly and acting as an enterotoxin capable of inducing diarrhea [54,55]. On the other hand, the supplementation with 2’-FL and scGOS/lcFOS/2’-FL increased the levels of TLR5 and TLR7 compared to both the RV and REF groups. These innate receptors are involved in the sensing of bacterial flagellin and ssRNA, respectively. TLR5 is crucial in the sensing of commensal bacteria, and its loss has shown to alter the normal gut microbiome leading to a dysbiosis characterized by chronic intestinal inflammation and outgrowth of *Proteobacteria*, far more motile than normal and with a high source of pro-inflammatory flagellin in the gut [56]. In this context, the increase of TLR5 displayed by 2’-FL and scGOS/lcFOS/2’-FL supplementations at the peak of RV infection would suggest a higher resistance to dysbiosis, a fact which was also supported by the preventive ability in the colonization of many genera of the *Proteobacteria* phylum that appeared in the RV group. Moreover, it is suggested that the activation of TLR5/NLRC4 is involved in the cure and prevention of RV infection [57] and that mRNA levels of TLR5 do not seem to be changed by the absence or presence of gut microbiota [58]. The rise in TLR7 expression would also mean a higher antiviral state, as TLR7 has been shown to trigger interferon (IFN)α/β expression [59]. Indeed, such increase could indicate a better sensing and response against RV, which although it is a dsRNA virus, some studies suggest that its genetic material can be degraded towards ssRNA in the endosomal compartment and, therefore, can be recognized by TLR7 [59,60,61] (Figure 7).

It would be interesting to know whether, besides these changes in TLR gene expression, a direct interaction of the tested oligosaccharides with the TLR or even with host lectins is produced. The microbiota composition in the human neonatal gut is mainly driven by the host environment, delivery mode (vaginal/caesarian), and type of feeding (breast/formula) [62]. This microbial population changes rapidly over time until 2 to 3 years of age, when it stabilizes towards an adult-like configuration [63]. In the first week of life, microbiota is frequently dominated by facultative anaerobes, which consume oxygen, and thus afterwards, they make the habitat suitable for the colonization by strict anaerobes [64]. This principle of succession in rodents resembles that in humans, although it shows a differential pattern. Both are vastly colonized by *Escherichia* and *Streptococcus* but, while humans display more *Bifidobacterium*, rodents bias towards more *Lactobacillus* [65,66]. In the present study, the microbiota of the 8-day neonatal rat was dominated by *Firmicutes* (40%), *Proteobacteria* (35%), and *Actinobacteria* (23%), being *Lactobacillus*, *Escherichia*, and *Rothia*, respectively, and the genera represented more than 90% of the microbiota.

The main limitation of this study is the low number of samples analyzed for microbial composition determination; that is why these interesting results should be carefully interpreted and taken into account as a first approach for the dysbiosis caused by the RV and the effect of the oligosaccharides tested here. In addition, the V3–V4 sequencing used here can also be improved in future studies by using a metagenomic approach. Overall, based on these results, the infection with RV induced a dysbiosis mainly by decreasing normally residing members, such as *Streptococcus* and *Staphylococcus*, although it did not affect bacterial diversity. These findings are in line with a study of Chen et al. [12], which showed a reduced Shannon Index only in severe but not mild, acute gastroenteritis in patients suffering from RV infection. In the same context, Li et al. [10] found decreased Shannon Index and CHAO1 when infecting piglets with an homologous RV strain. It is well-known that an imbalanced gut microbiota often arises from increases in *Proteobacteria*, which have been associated with a higher inflammatory state and several diseases, such as colitis and metabolic syndrome [64,67]. This increase is also present in the case of the RV group; in fact, some *Proteobacteria* that appeared have been attributed to inflammatory properties, such as *Klebsiella* and *Serratia*, which are associated with necrotizing enterocolitis and sepsis in premature infants [68]. When performing the PCA, it was striking that the bacterial composition of those animals in the RV group clustered apart from the other individuals, suggesting that RV infection evidently disturbed the normal microbiota composition.

The neonatal rat is a suitable model to assess the effects of the assayed oligosaccharides, such as 2’-FL and the mixture of scGOS/lcFOS. The first is globally one of the main oligosaccharides in human milk, but it is not so abundant in rat milk, which is dominated mainly by 3’-sialyllactose (3’-SL) [69]. The well-studied prebiotic mixture of scGOS/lcFOS is produced from milk and vegetable sources and induced similar microbial profiles as detected with infants receiving human milk [70]. The daily nutritional intervention with these oligosaccharides was able to prevent the dysbiosis induced by the RV infection (Figure 7).

It has been described that fucosylated human milk oligosaccharides, such as 2’-FL, may be involved in the decrease of intestinal *Proteobacteria*, particularly in *Enterobacteriaceae* [31,68], which is in line with the prevention of *Proteobacteria*-associated dysbiosis found here. Conversely, it has been reported a positive correlation for 2’-FL and fecal bacteria from *Firmicutes* phylum [31,68]. In our study, although the supplementation with 2’-FL did not show differences in the abundance of the *Firmicutes* population, we observed that it promoted the growth of several genera (e.g., *Staphylococcus* and *Turicibacter*) and the colonization of many new members, with a notable importance of nonpathogenic *Clostridium*. Most of the *Clostridium* that appeared belonged to the clusters IV (e.g. *C. leptum*) and XIVa (e.g. *C. asparagiforme* and *C. lavalense*), of which cultivated members are well-known for their health-promoting activities arisen from the production of butyric acid, with anti-inflammatory and immunomodulatory properties [71]. Moreover, *Roseburia* and *Ruminococcus*, genera included in the *Clostridium* cluster XIVa, also appeared after the supplementation with 2’-FL. Several studies report a depletion of butyrogenic bacteria belonging to clusters IV and XIVa in diseases coursing with dysbiosis, including diarrhea [71,72,73,74]. In addition, it has been described that individuals with a greater abundance of *Ruminococcaceae* might have lower susceptibility to RV infection [13].

Some of the effects displayed after the supplementation with 2’-FL were also seen by scGOS/lcFOS, such as the colonization with *Roseburia* and *Ruminococcus* or the higher abundance of *Staphylococcus* compared to the RV group. However, scGOS/lcFOS supplementation induced other specific effects, such as promoting the colonization of 9 species-like groups within the *Lactobacillus* genus (e.g., *L. gasseri* and *L. rhamnosus*). The combination of scGOS/lcFOS and 2’-FL displayed additive effects because it promoted the colonization of butyrate-producing bacteria and prevented *Streptococcus* and *Staphylococcus* decrease and the colonization of *Proteobacteria*.

The overall microbiota structure altering effects of these oligosaccharides were confirmed with the PCA, which allowed seeing that the three supplements caused a different microbiota composition compared to the RV group, suggesting that the beneficial effects for the prevention of the dysbiosis occurred. In addition, the microbiota from the animals receiving oligosaccharides was more similar to that of the REF group.

The data herein also suggested that many bacteria with probiotic characteristics are present in higher abundances in individuals administered with oligosaccharides. In this sense, both dietary interventions have showed a tendency to increase the abundance of bifidobacteria; however, this effect has not been significantly evidenced maybe due to the dysbiosis caused by the RV and the low levels of initial bifidobacteria present in suckling rats. Nevertheless, the growth promoting effect of scGOS/lcFOS and 2’-FL in humans has been documented [31,32,33]. In infants, 2’-FL is shown to increase the gut-predominant *Bifidobacterium infantis* and *Bifidobacterium bifidum* [75,76]. However, in suckling rats, the abundance of *Bifidobacterium* is much lower and the species which are present (e.g. *B. animalis* and *B. thermophilum*) do not use 2’-FL as a source of energy [76]. Thus, the potential growth of these beneficial bacteria by 2’-FL remains unclear, as it does not seem to be related to a direct 2’-FL catabolism [77]. In contrast, the higher abundance of probiotic bacteria might be linked to the catabolism of scGOS/lcFOS, which has been shown to be a good energy source for many of the bacteria identified [76,78,79]. However, the growth promoting effect of scGOS/lcFOS on *Lactobacillus rhamnosus* and *Bifidobcterium animalis* did not appear when it was combined with 2’-FL, a fact that may suggest that 2’-FL may be interfering somehow in the prebiotic effect of scGOS/lcFOS.

Finally, it would be interesting to study the role of these oligosaccharides in the context of an anti-RV vaccination process in which their action preventing the dysbiosis induced by the virus could have and additional value. In this line, they can be even more beneficial in those cases of severe RV infection in which fecal microbiota transplantation could be an option either alone or in combination with these components.

## 5. Conclusions

Despite the small sample size used in the present study, this first approach studying the effect of certain oligosaccharides on microbiota composition supports a preventive effect of scGOS/lcFOS and 2’-FL in the RV diarrhea in rats, which could be associated with changes in the microbiota–host crosstalk and the microbiota composition. Moreover, the combination of scGOS/lcFOS and 2’-FL maintains the specific effects of each compound by providing this mixture with higher benefits than those induced by the compounds separately.

The infection with RV seemed to induce intestinal dysbiosis in terms of reducing normal residing bacterial populations, such as *Streptococcus* and *Staphylococcus*, and of promoting the colonization with many *Proteobacteria* with inflammatory potential. RV dysbiosis seemed successfully prevented by the administration of all oligosaccharides, which in addition promoted a healthy microbiota comparable to noninfected rats. Therefore, the supplementation with these oligosaccharides in early life, for example, by adding them into infant formulas, seems to be a good strategy to prevent or treat RV infection and its consequences.

## Figures and Tables

**Table 1:** Overall diversity of fecal microbiota at the peak of diarrhea (day 8).

	REF	RV	RV+scGOS/lcFOS	RV+2’-FL	RV+scGOS/lcFOS/2’-FL
**Number of sequences**	39248 ± 2944	38902 ± 982	41732 ± 1528	31028 ± 3510	36070 ± 1703
**Number of OTUs**	103 ± 9	113 ± 12	124 ± 9	143 ± 39	104 ± 22
**Shannon Index**	1.68 ± 0.08	1.61 ± 0.07	1.74 ± 0.05	1.92 ± 0.17	1.61 ± 0.04
**CHAO1 Richness**	259.7 ± 50.2	285.0 ± 60.4	251.7 ± 7.9	263.0 ± 46.0	187.0 ± 46.5

16S rRNA gene v3–v4 was sequenced by the Metagenomic Sequencing Library Illumina 15044223 B protocol. The Shannon Index and CHAO1 estimator were calculated according to the Operational Taxonomic Units (OTU) numbers of each group. Results are expressed as mean ± S.E.M. (n = 3/group). No statistical differences were found (by the Mann-Whitney U test).

## Supplementary Materials

The following are available online at https://www.mdpi.com/2073-4409/8/8/876/s1, Figure S1: Summary of species variation in fecal microbiota at the peak of diarrhea (day 8).
