# Supplementary material for: Oligosaccharides Modulate Rotavirus-Associated Dysbiosis and TLR Gene Expression in Neonatal Rats
Source: Cells. 2019 Aug 11;8(8):876. doi: 10.3390/cells8080876 (PMC6721706; doi:10.3390/cells8080876)
Supplement: Supplementary file 1 [file cells-08-00876-s001.pdf]

cells

MDPI

SUPPLEMENTARY FIGURE 1

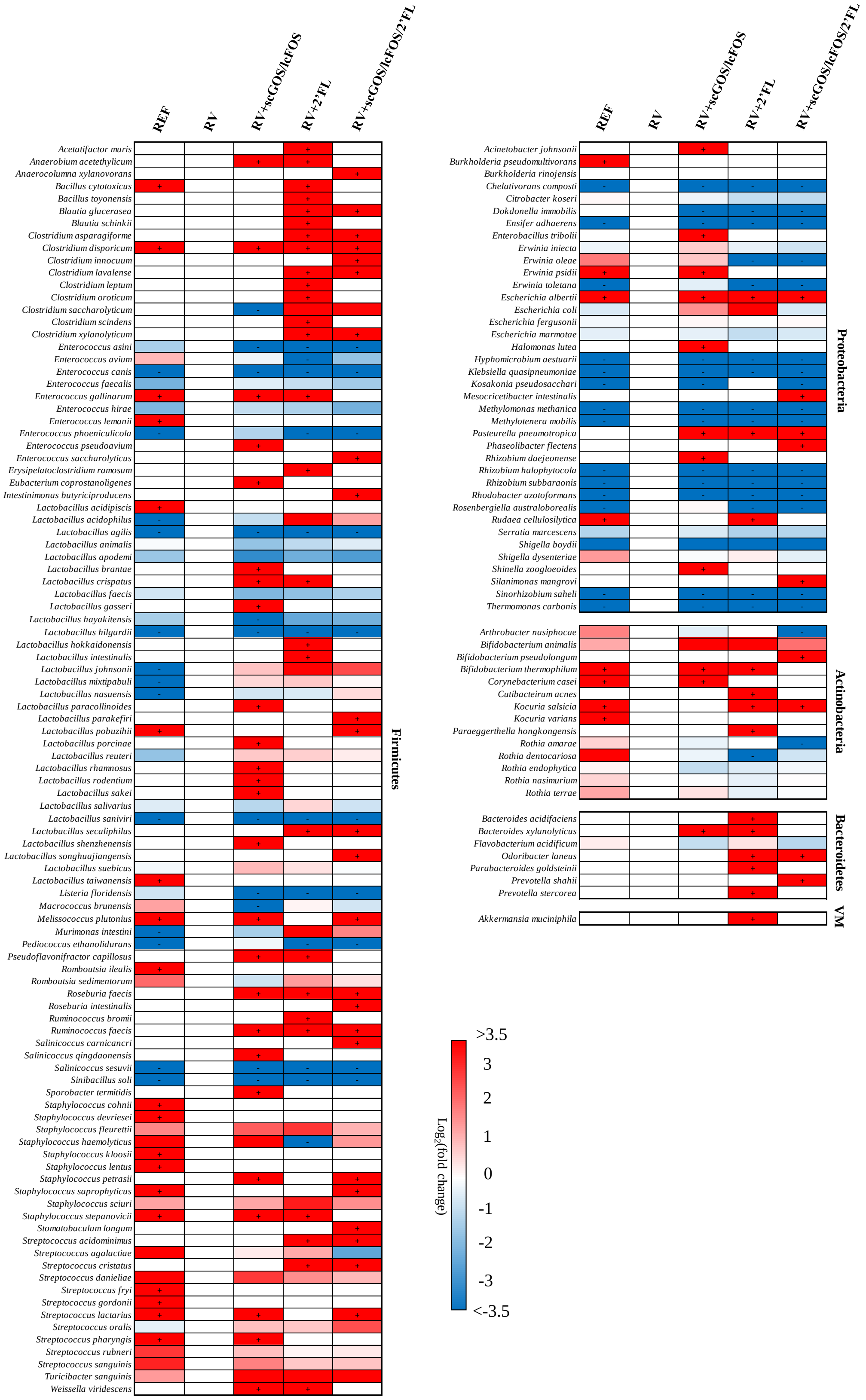

Supplementary Figure 1. Summary of species variation in fecal microbiota at the peak of diarrhea (day 8). A heat map of the mean relative abundances of the prominent OTUs assigned to the species level is represented. The rows represent the species and the columns the experimental groups. The log_2 of the fold change in respect to RV group was calculated and assigned a color following the legend. Species which were present in a group but not in the RV group were assigned the maximum variation (>3.5, marked with a "+"), whereas the genera which were present in RV group and not in the other groups were assigned the minimum variation (<-3.5, marked with a "-"). Results derived from n=3/group. VM: *Verrucomicrobia*.

The RV group showed higher abundance of *Lactobacillus* species (e.g. *Lactobacillus salivarius*, p<0.05) than the REF group. Moreover, the infection decreased or excluded 7 species of *Staphylococcus* and 8 of *Streptococcus*. The supplementations partially counteracted some of these effects. scGOS/lcFOS induced the colonization of 9 new species of *Lactobacillus*, which were different from those present in the RV group. Moreover, the alteration of *Staphylococcus* and *Streptococcus* species found in RV group was not so apparent. In fact, scGOS/lcFOS supplementation prevented the exclusion of 3 species whereas enhanced the levels of 7 others to abundances similar as those in the REF group (e.g. *Staphylococcus haemolyticus* and *Streptococcus danieliae*). The supplementation with 2'FL promoted the colonization of several bacteria of the *Firmicutes* phyla, such as 7 different *Clostridium* species and 2 *Bacillus* species. The alteration of *Staphylococcus* and *Streptococcus* species was again not as intense as in the RV group. In addition, the RV infection promoted the colonization of numerous species belonging to the *Actinobacteria* and *Bacteroidetes* phyla. The mixture of scGOS/lcFOS and 2'FL seemed to combine the effects found separately for these oligosaccharides and interestingly, all oligosaccharides seemed to prevent the colonization of *Listeria floridensis*. Finally, the three supplementations were found to block the colonization of many species from the *Proteobacteria* phylum compared to the RV group (e.g. *Klebsiella quasipneumoniae* and *Rhodobacter azotoformans*), making them more similar to the REF group.
